# Otosyphilis: A rare cause of acute bilateral sensorineural hearing loss in a HIV-negative patient

**DOI:** 10.4102/sajr.v26i1.2351

**Published:** 2022-03-29

**Authors:** Johan Sothmann, Shaun Adam, Gideon van Tonder, Razaan Davis, Leon Janse van Rensburg

**Affiliations:** 1 Department of Medical Imaging and Clinical Oncology, Faculty of Medicine and Health Sciences, Stellenbosch University, Cape Town, South Africa; 2 Division of Otorhinolaryngology, Faculty of Medicine and Health Sciences, Stellenbosch University, Cape Town, South Africa; 3 Department of Radiology and Diagnostics, Faculty of Dentistry, University of the Western Cape, Cape Town, South Africa

**Keywords:** otosyphilis, sensorineural, computed tomography, magnetic resonance imaging, audiology, human immunodeficiency virus (HIV)

## Abstract

Bilateral acute hearing loss is rare, and the aetiology is poorly defined. Less common treatable pathologies such as otosyphilis must be part of the differential diagnosis and should be actively excluded. We present a case of a 23-year-old woman who developed acute bilateral hearing loss due to otosyphilis, confirmed on audiometry and laboratory tests. In this article, the CT, MRI and clinical findings are presented and discussed.

## Introduction

Hearing loss is categorised as sensorineural, conductive or mixed. The differential diagnosis for each category is wide, and the combination of clinical evaluation, laboratory findings and imaging features are important in determining the diagnosis. In many patients, an underlying pathology is not found with a consequent diagnosis of idiopathic sudden hearing loss.^1^ Bilateral acute hearing loss is rare and occurs in approximately 1% – 2% of cases.^2^ The aetiology is poorly defined and includes infective, vascular and neoplastic aetiologies.^3^ It is important in everyday practice to consider less common but treatable pathologies such as syphilis.

## Case presentation

A 23-year-old woman presented to a local emergency centre complaining of sudden onset of left-sided otalgia, headache and tinnitus two weeks prior. This was followed by the development of left facial weakness and sudden bilateral hearing loss a week before her presentation. There was no history of prior ear disease or surgery and no other medical illnesses.

Clinical examination revealed left lower motor neurone facial nerve paresis (House-Brackmann grade 5), bilateral sensorineural hearing loss, a positive catch-up saccade on head impulse test and a macular rash on her torso and chest. There were no other neurological deficits or ear, nose and throat (ENT) abnormalities and the gynaecological examination was normal. An empirical treatment regime consisting of ceftriaxone, prednisone and acyclovir was started.

### Audiometry

**FIGURE 1:**
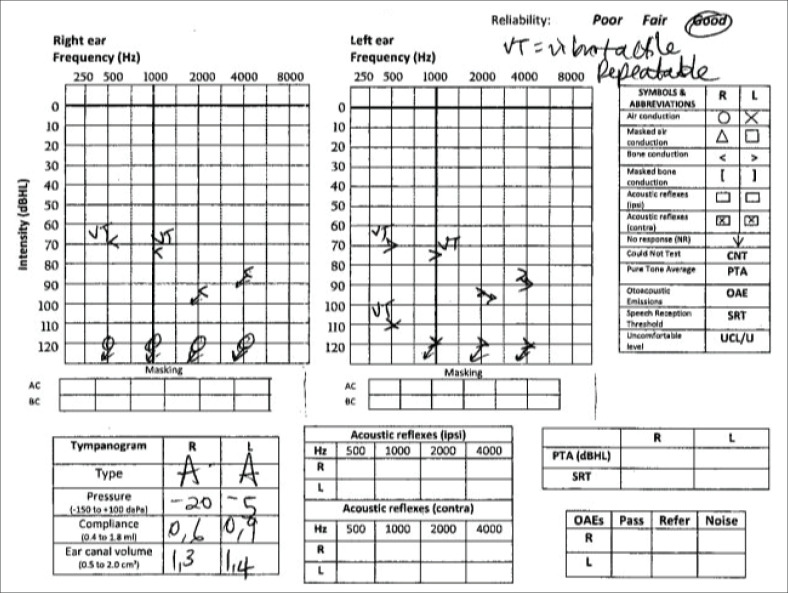
Audiogram at the time of admission demonstrated bilateral profound sensorineural hearing loss.

The audiogram confirmed bilateral severe-to-profound sensorineural hearing loss with type A tympanometry (Figure 1).

### Computed Tomography and Magnetic Resonance Imaging

**FIGURE 2:**
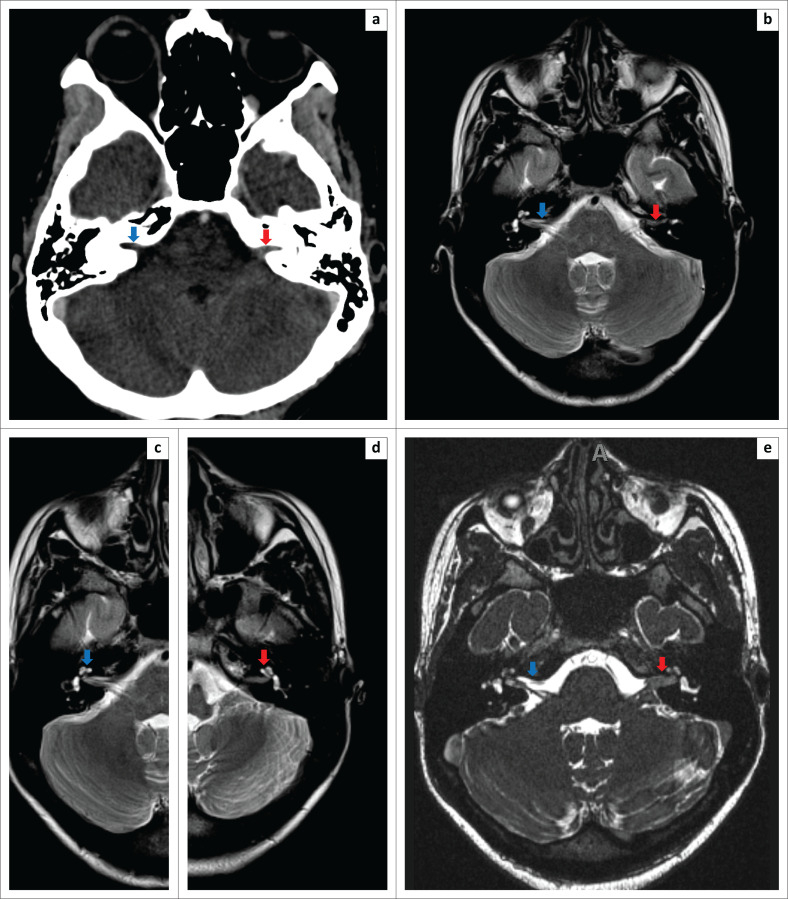
(a) Axial CT brain scan demonstrated a hyperattenuating mass at the porous acousticus of the left internal acoustic canal (red arrow). There was mild contrast enhancement and the canal was not expanded. The right internal acoustic canal appeared normal (blue arrow). (b) Axial, T2W MRI of the IAC demonstrated loss of the normal CSF signal within the left IAC (red arrow). The right IAC demonstrated signal loss to a lesser extent (blue arrow). (c–d) Axial, T2W, MRI of the cochleae demonstrated partial loss of the normal CSF signal in the basal turns of the cochleae bilaterally (blue arrow in c indicating the right and red arrow in d indicating the left cochlea). (e) Axial, MRI, constructive interference in steady state sequence, demonstrated a notable mass within the left internal acoustic canal, with loss of the normal CSF signal (red arrow). This was seen to a lesser extent in the right internal acoustic canal (blue arrow).

A contrasted CT brain scan demonstrated a hyperattenuating mass at the porous acousticus of the left internal acoustic canal (IAC) (Figure 2a). There was mild contrast enhancement and the canal was not expanded. The right IAC appeared normal. The provisional diagnosis was a left-sided vestibular schwannoma.

Magnetic resonance imaging (MRI) of the brain and IACs with intravenous gadolinium was performed. This demonstrated loss of the normal CSF signal within the left IAC on T2-weighted (T2W) imaging. The right IAC demonstrated signal loss to a lesser extent (Figure 2b). There was partial loss of the normal CSF signal in the basal turns of the cochleae bilaterally (Figure 2c and d).

The three-dimensional constructive interference in steady state (CISS) revealed a hypointense mass within the left IAC, with loss of the normal CSF signal sequence. Less obvious changes were present in the right IAC (Figure 2e).

**FIGURE 3:**
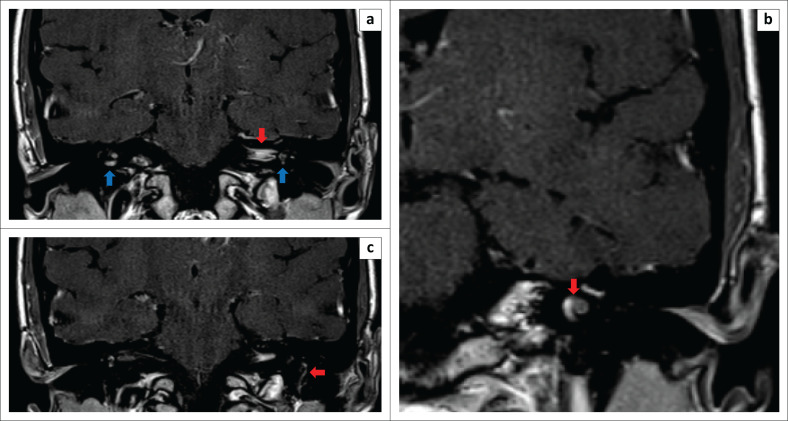
(a) Coronal, T1 weighted post contrast MRI demonstrated enhancement in left internal acoustic canal and facial nerve (red arrow) as well as bilateral basal turns of the cochleae (blue arrows). (b) Coronal, T1 weighted post contrast MRI demonstrated enhancement of bilateral basal turns of the cochleae (only left side shown, indicated by red arrow). (c) Coronal, T1 weighted post contrast MRI demonstrated enhancement of the left 7th cranial nerve vertical segment with a nodular appearance (red arrow).

On T1-weighted (T1W) imaging the right-sided IAC mass was isointense to brain parenchyma. T1-weighted post contrast imaging revealed vivid contrast enhancement in the left IAC and facial nerve (Figure 3a), as well as in the basal turns of the cochleae bilaterally (Figure 3b). Leptomeningeal enhancement anterior to the pons as well as the outline of the IAC was demonstrated. The vertical segment of the left seventh cranial nerve (CN) demonstrated a nodular pattern of contrast enhancement (Figure 3c).

### Laboratory findings

Serum tests indicated an elevated C-reactive protein (CRP) 14 mg/L and an erythrocyte sedimentation rate (ESR) of 28 mm/h. The HIV status of the patient was negative. The white blood cell count, renal and thyroid function tests were normal. The serum *Treponema pallidum* haemagglutination (TPHA) assay test and the rapid plasma reagin (RPR) were positive with an initial RPR titre of 1:128. Serum viral studies for cytomegalovirus (CMV), Epstein barr virus (EBV), mumps, enterovirus, herpes simplex virus 1 and 2, and human herpes virus type 6, were all negative.

The lumbar puncture revealed a normal glucose level, mildly elevated protein, elevated lymphocytes, no bacteria nor bacterial growth and negative cryptococcal and viral panel tests. The fluorescent *Treponema pallidum* antibody test (FTA-ABS) was positive. Unfortunately, the venereal disease research laboratory (VDRL) test on CSF was not available.

Sputum gene Xpert was negative. The severe acute respiratory syndrome coronavirus 2 (SARS‑CoV‑2) swab was negative.

### Management

Positive treponema pallidum serology with a high RPR titre (1:128) that reduced fourfold on treatment, confirmed current infection. The findings of CSF with positive FTA confirmed CNS involvement with secondary syphilis. The audiological findings, clinical and radiological picture, together with the absence of another cause, confirmed otosyphilis in this case. Initial ceftriaxone was changed to intravenous penicillin G 6 million units 6 hourly for 14 days, together with daily oral 1 mg/kg prednisone and eye care to prevent exposure keratitis due to her facial nerve palsy.

Repeat audiometry demonstrated no improvement in the hearing loss, but her facial nerve function improved to near normal (House-Brackmann 1). Her speech discrimination score was 0% bilaterally so she would not have derived any benefit from a hearing aid. Her current social circumstances unfortunately preclude her candidacy for a cochlear implant at this time. She has been referred to the National Institute for the Deaf to learn sign language and for occupational training so she may be upskilled and obtain employment.

## Discussion

Otosyphylis (syphilitic labyrinthitis) is a manifestation of neurosyphilis affecting the inner ear. The radiological features include severe endolymphatic hydrops, membranous labyrinth degeneration and diffuse osteitis.^4^ Histopathological features of otosyphilis are secondary to obliterative endarteritis and lead to bone involvement with inflammatory resorptive osteitis and areas of resorption. The CT correlate of this process is otocapsule permeative and irregular lucencies, close to the labyrinth margins. Lucencies within the malleus and incus may also be present.^5^ MRI features may include gummas in the internal auditory canal, as well as other manifestations of neurosyphilis, which include leptomeningeal enhancement, CN 7 and 8 enhancement, cortical and subcortical infarctions, and also segmental vascular narrowing and beading (best seen on angiography). Gummas appear as small nodules adjacent to the meninges which are T1W hypointense and T2W hyperintense, and demonstrate homogenous contrast enhancement. Gummas may also demonstrate a dural tail sign or evidence of pachymeningitis. The MRI findings of late neurosyphilis may reveal cerebral and cord atrophy.^6^

The differential diagnosis based on the MRI findings should include Ramsey Hunt Syndrome (Herpes Zoster Oticus) and acoustic schwannoma, as the MRI findings in these entities may overlap.^7,8^ Serology and CSF findings are, therefore, crucial to attribute the MRI findings to neurosyphilis.

In this patient, the MRI features of acute-phase neurosy philis were present, without any radiographic features of osteitis and osteolysis of the adjacent temporal bone. The pertinent radiological findings were bilateral IAC gadolinium contrast agent enhancement with a mass in the left IAC, involvement of both inner ears with predominance on the left and a small T1W hyperintense focus in the basal turn consistent with proteinaceous content or a gumma, and post-contrast leptomeningeal and bilateral facial nerve enhancement. These findings were concordant with previous reports and interpreted as a result of the spread of the inflammatory process via the endolymphatic and/or the perilymphatic fluids. The diagnosis of bilateral otosyphilis with early syphilitic meningitis and bilateral vestibulocochlear and facial nerve neuritis was made.

A high-resolution temporal bone CT was performed six weeks post-treatment, which revealed normal bilateral temporal bones. Repeat MRI was unchanged. This case highlights the importance of an expeditious and correct diagnosis by a multidisciplinary team as early and prompt treatment initiation may reverse the hearing loss.^9^ Outcome is improved for younger patients, and also if unilateral hearing loss is present for a shorter period before the diagnosis. The outcome is generally poorer in bilateral or in complete hearing loss,^1^ as in the presented case.

Syphilis affects over 30 million people across the globe. Primary syphylis occurs at the inoculation site and may present as a painless macule. Secondary syphylis usually occurs 4–8 weeks later and may present with a rash involving the hands and feet. If left untreated, tertiary syphylis will develop in approximately 30% of cases and mainly manifests as cardiovascular and neurosyhylis. Neurological involvement may present in both the early and late stages of the disease.^10^

Most patients diagnosed with otosyphylis are usually co-infected with HIV.^11^ Otosyphyllis in a HIV-negative patient is thought to be rare, and there are limited recent available data on this subject.

Eastern and Southern Africa is the region in the world most affected by HIV.^12^ The risk of being infected with syphilis is increased in the HIV-infected patient and the progression of disease is also faster.^13^ Both syphylis and HIV have the highest prevalence in Africa, and it would therefore be expected that neurosyphyllis would not be an uncommon manifestation of syphilis in Africa.^14^ Otosyphyllis is one of the clinical manifestations of neurosyphylis,^15^ and clinicians should have a high index of suspicion in patients who are at risk, particularly HIV-infected patients, who present with acute auditory and vestibular symptoms.

The rising incidence of syphilis in the past two decades^1,9^ underscores the importance of including otosyphilis as part of the differential diagnosis for acute bilateral hearing loss, regardless of the HIV status.

## Conclusion

An accurate and definitive diagnosis of otosyphilis remains challenging and should be based on clinical imaging and laboratory (serological and CSF) findings.^7^ The presented case demonstrates how imaging findings, correlated with clinical findings and laboratory results, assisted in confirmation of the diagnosis and determining the extent of the disease.
